# 2-Hy­droxy­iso­quinoline-1,3(2*H*,4*H*)-dione

**DOI:** 10.1107/S1600536813019843

**Published:** 2013-07-24

**Authors:** Yoshinobu Ishikawa, Soichiro Matsuo

**Affiliations:** a School of Pharmaceutical Sciences, University of Shizuoka, 52-1 Yada, Suruga-ku, Shizuoka 422-8526, Japan

## Abstract

The title mol­ecule, C_9_H_7_NO_3_, exists in the diketo form and the iso­quinoline unit is approximately planar (r.m.s. deviation = 0.0158 Å). In the crystal, mol­ecules are linked into inversion dimers through pairs of O—H⋯O hydrogen bonds and are further assembled into the (100) layers *via* stacking inter­actions [centroid–centroid distances = 3.460 (3) and 3.635 (4) Å].

## Related literature

For the biological properties of the title compound, see: Parkes *et al.* (2003 ▶); Hang *et al.* (2004 ▶); Billamboz *et al.* (2008 ▶). For a related structure, see: Miao *et al.* (1995 ▶).
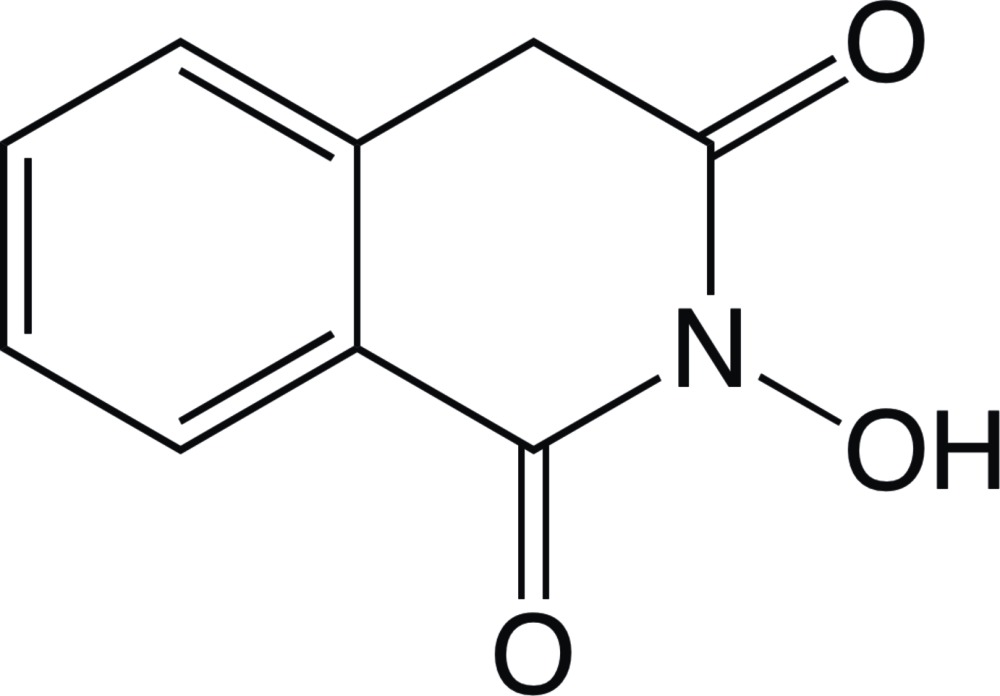


## Experimental

### 

#### Crystal data

C_9_H_7_NO_3_
*M* *_r_* = 177.16
Monoclinic, 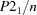

*a* = 12.336 (5) Å
*b* = 8.666 (4) Å
*c* = 7.052 (7) Å
β = 104.19 (5)°
*V* = 730.8 (9) Å^3^
*Z* = 4
Mo *K*α radiation
μ = 0.12 mm^−1^
*T* = 100 K
0.50 × 0.50 × 0.45 mm

#### Data collection

Rigaku AFC-7R diffractometer
3873 measured reflections
1650 independent reflections
1484 reflections with *F* ^2^ > 2σ(*F* ^2^)
*R*_int_ = 0.016
3 standard reflections every 150 reflections intensity decay: −0.5%

#### Refinement

*R*[*F* ^2^ > 2σ(*F* ^2^)] = 0.032
*wR*(*F* ^2^) = 0.089
*S* = 1.04
1650 reflections
119 parameters
H-atom parameters constrained
Δρ_max_ = 0.25 e Å^−3^
Δρ_min_ = −0.25 e Å^−3^

### 

Data collection: *WinAFC Diffractometer Control Software* (Rigaku, 1999 ▶); cell refinement: *WinAFC Diffractometer Control Software*; data reduction: *WinAFC Diffractometer Control Software*; program(s) used to solve structure: *SHELXS97* (Sheldrick, 2008 ▶); program(s) used to refine structure: *SHELXL97* (Sheldrick, 2008 ▶); molecular graphics: *CrystalStructure* (Rigaku, 2010 ▶); software used to prepare material for publication: *CrystalStructure*.

## Supplementary Material

Crystal structure: contains datablock(s) global, I. DOI: 10.1107/S1600536813019843/gk2583sup1.cif

Structure factors: contains datablock(s) I. DOI: 10.1107/S1600536813019843/gk2583Isup2.hkl

Supplementary material file. DOI: 10.1107/S1600536813019843/gk2583Isup3.cml

Additional supplementary materials:  crystallographic information; 3D view; checkCIF report

## Figures and Tables

**Table 1:** Hydrogen-bond geometry (Å, °)

*D*—H⋯*A*	*D*—H	H⋯*A*	*D*⋯*A*	*D*—H⋯*A*
O5—H5⋯O1^i^	0.84	1.91	2.7056 (17)	158

Symmetry code: (i) 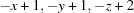.

**Fig. 1.**
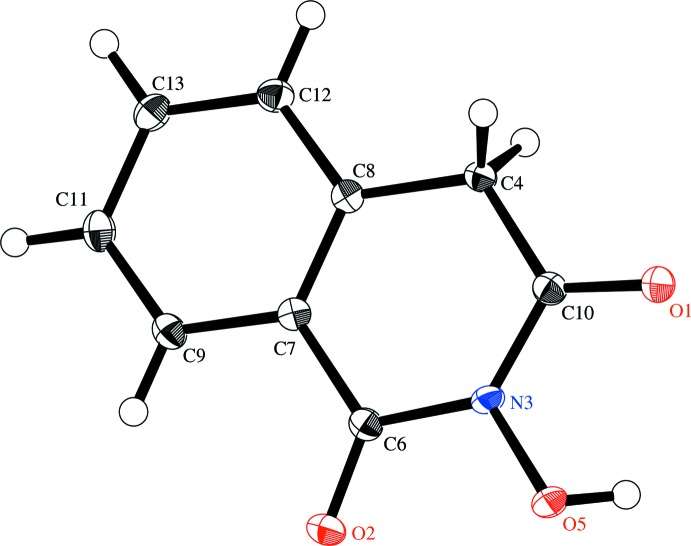
The molecular structure of the title compound, with displacement ellipsoids drawn at the 50% probability level. Hydrogen atoms are shown as small spheres of arbitrary radius.

**Fig. 2.**
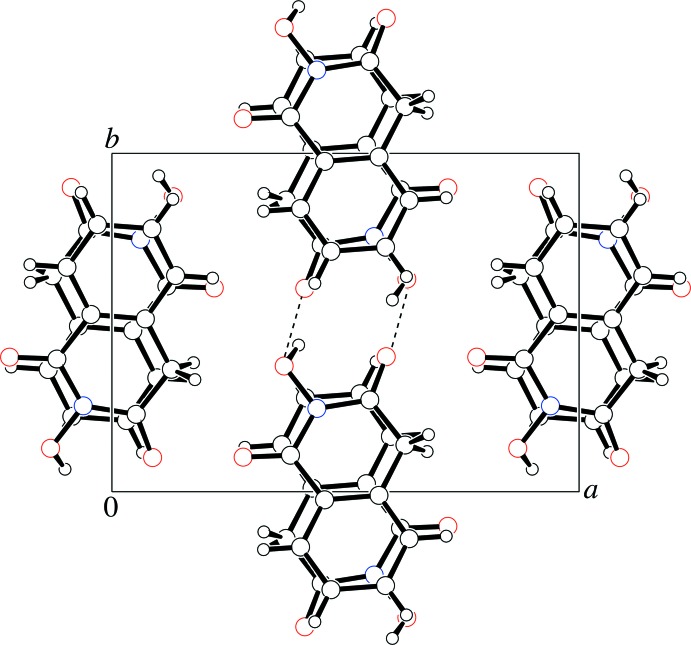
A crystal packing view of the title compound. Hydrogen bonds are represented as dashed lines.

## supplementary crystallographic information

### Comment

The title compound is known to inhibit metalloenzymes such as influenza endonuclease (Parkes *et al.*, 2003), HIV-1 reverse transcriptase RNase H (Hang *et al.*, 2004), and HIV-1 integrase (Billamboz *et al.*, 2008). Here we report the crystal structure of the title compound, which was obtained from the deprotection of 2-benzyloxyisoquinoline-1,3(2*H*,4*H*)-dione by the use of boron tribromide. The compound exists in keto form and the isoquinoline ring is almost planar (r.m.s. deviation = 0.0158 Å). In the crystal, the molecules link through intermolecular O–H···O hydrogen bonds and stack along the *c* axis, as shown in Figure 2. The distance from plane1 (C7/C8/C9/C11/C12/C13) to plane2 [C4/C6/C7/C8/C10/N3, (1 - *x*, 2 - *y*, 1 - *z*)] is 3.460 (3) Å.

### Experimental

The title compound was synthesized according to the literature (Billamboz *et al.*, 2008). Single crystals suitable for X-ray diffraction were obtained by slow evaporation of an acetone solution of the compound at room temperature.

### Refinement

The hydrogen atoms of the benzene ring were placed geometrically [C–H 0.95 Å, *U*_iso_(H) = 1.2*U*_eq_(C)], and refined using a riding model. The hydrogen atoms of the methylene and N–OH groups were found in a difference Fourier map, and refined with distance constraints [C–H 0.99 Å, *U*_iso_(H) = 1.2*U*_eq_(C), O–H 0.84 Å, *U*_iso_(H) = 1.2*U*_eq_(O)].

### Crystal data

**Table:**

C_9_H_7_NO_3_	*F*(000) = 368.00
*M**_r_* = 177.16	*D*_x_ = 1.610 Mg m^−^^3^
Monoclinic, *P*2_1_/*n*	Mo *K*α radiation, λ = 0.71069 Å
Hall symbol: -P 2yn	Cell parameters from 25 reflections
*a* = 12.336 (5) Å	θ = 14.9–17.0°
*b* = 8.666 (4) Å	µ = 0.12 mm^−^^1^
*c* = 7.052 (7) Å	*T* = 100 K
β = 104.19 (5)°	Block, orange
*V* = 730.8 (9) Å^3^	0.50 × 0.50 × 0.45 mm
*Z* = 4	

### Data collection

**Table:**

Rigaku AFC-7R diffractometer	θ_max_ = 27.5°
ω–2θ scans	*h* = −16→15
3873 measured reflections	*k* = −11→11
1650 independent reflections	*l* = −5→9
1484 reflections with *F*^2^ > 2σ(*F*^2^)	3 standard reflections every 150 reflections
*R*_int_ = 0.016	intensity decay: −0.5%

### Refinement

**Table:**

Refinement on *F*^2^	Secondary atom site location: difference Fourier map
*R*[*F*^2^ > 2σ(*F*^2^)] = 0.032	Hydrogen site location: inferred from neighbouring sites
*wR*(*F*^2^) = 0.089	H-atom parameters constrained
*S* = 1.04	*w* = 1/[σ^2^(*F*_o_^2^) + (0.0517*P*)^2^ + 0.2377*P*] where *P* = (*F*_o_^2^ + 2*F*_c_^2^)/3
1650 reflections	(Δ/σ)_max_ < 0.001
119 parameters	Δρ_max_ = 0.25 e Å^−^^3^
0 restraints	Δρ_min_ = −0.25 e Å^−^^3^
Primary atom site location: structure-invariant direct methods	

### Special details

**Table:**

Refinement. Refinement was performed using all reflections. The weighted *R*-factor (*wR*) and goodness of fit (*S*) are based on *F*^2^. *R*-factor (gt) are based on *F*. The threshold expression of *F*^2^ > 2.0 σ(*F*^2^) is used only for calculating *R*-factor (gt).

### Fractional atomic coordinates and isotropic or equivalent isotropic displacement parameters (Å^2^)

**Table:**

	*x*	*y*	*z*	*U*_iso_*/*U*_eq_	
O1	0.41291 (6)	0.60097 (8)	0.84881 (11)	0.01900 (19)	
O2	0.71902 (6)	0.89780 (9)	0.89569 (11)	0.01901 (19)	
O5	0.63125 (6)	0.62843 (8)	0.92088 (12)	0.01860 (19)	
N3	0.56130 (7)	0.75393 (9)	0.86013 (12)	0.0136 (2)	
C4	0.37267 (8)	0.86193 (11)	0.74485 (15)	0.0132 (2)	
C6	0.61776 (8)	0.89219 (11)	0.84438 (14)	0.0133 (2)	
C7	0.54474 (8)	1.02389 (11)	0.76248 (13)	0.0124 (2)	
C8	0.42836 (8)	1.01072 (11)	0.71477 (13)	0.0124 (2)	
C9	0.59616 (8)	1.16334 (12)	0.73257 (14)	0.0149 (3)	
C10	0.44805 (8)	0.72873 (11)	0.82056 (14)	0.0134 (2)	
C11	0.53109 (9)	1.28959 (12)	0.65699 (15)	0.0164 (3)	
C12	0.36371 (8)	1.13890 (12)	0.63746 (15)	0.0151 (3)	
C13	0.41430 (9)	1.27737 (12)	0.60954 (15)	0.0165 (3)	
H4A	0.3253	0.8813	0.8372	0.0158*	
H4B	0.3224	0.8312	0.6184	0.0158*	
H5	0.6004	0.5666	0.9829	0.0223*	
H9	0.6754	1.1710	0.7641	0.0179*	
H11	0.5655	1.3843	0.6373	0.0197*	
H12	0.2845	1.1312	0.6037	0.0182*	
H13	0.3696	1.3641	0.5581	0.0198*	

### Atomic displacement parameters (Å^2^)

**Table:**

	*U*^11^	*U*^22^	*U*^33^	*U*^12^	*U*^13^	*U*^23^
O1	0.0159 (4)	0.0140 (4)	0.0257 (4)	−0.0012 (3)	0.0025 (3)	0.0038 (3)
O2	0.0111 (4)	0.0193 (4)	0.0255 (4)	0.0003 (3)	0.0023 (3)	0.0022 (3)
O5	0.0134 (4)	0.0132 (4)	0.0283 (5)	0.0044 (3)	0.0034 (3)	0.0058 (3)
N3	0.0114 (4)	0.0112 (4)	0.0173 (4)	0.0027 (3)	0.0014 (3)	0.0018 (3)
C4	0.0104 (5)	0.0130 (5)	0.0158 (5)	0.0001 (4)	0.0028 (4)	0.0006 (4)
C6	0.0126 (5)	0.0142 (5)	0.0132 (5)	−0.0007 (4)	0.0032 (4)	−0.0011 (4)
C7	0.0133 (5)	0.0128 (5)	0.0112 (5)	0.0003 (4)	0.0030 (4)	−0.0012 (4)
C8	0.0132 (5)	0.0127 (5)	0.0116 (5)	0.0001 (4)	0.0039 (4)	−0.0012 (4)
C9	0.0140 (5)	0.0162 (5)	0.0146 (5)	−0.0028 (4)	0.0036 (4)	−0.0015 (4)
C10	0.0131 (5)	0.0144 (5)	0.0124 (5)	−0.0007 (4)	0.0027 (4)	−0.0011 (4)
C11	0.0198 (5)	0.0125 (5)	0.0176 (5)	−0.0029 (4)	0.0059 (4)	−0.0012 (4)
C12	0.0135 (5)	0.0154 (5)	0.0168 (5)	0.0019 (4)	0.0040 (4)	−0.0006 (4)
C13	0.0187 (5)	0.0129 (5)	0.0180 (5)	0.0030 (4)	0.0045 (4)	0.0004 (4)

### Geometric parameters (Å, º)

**Table:**

O1—C10	1.2230 (13)	C9—C11	1.3841 (15)
O2—C6	1.2133 (13)	C11—C13	1.4009 (17)
O5—N3	1.3891 (12)	C12—C13	1.3887 (16)
N3—C6	1.4039 (14)	O5—H5	0.840
N3—C10	1.3734 (14)	C4—H4A	0.990
C4—C8	1.5003 (15)	C4—H4B	0.990
C4—C10	1.4962 (15)	C9—H9	0.950
C6—C7	1.4807 (14)	C11—H11	0.950
C7—C8	1.3966 (15)	C12—H12	0.950
C7—C9	1.4046 (16)	C13—H13	0.950
C8—C12	1.3978 (15)		
			
O5—N3—C6	114.20 (9)	C9—C11—C13	119.84 (10)
O5—N3—C10	117.53 (8)	C8—C12—C13	120.58 (10)
C6—N3—C10	128.27 (8)	C11—C13—C12	120.20 (10)
C8—C4—C10	116.58 (9)	N3—O5—H5	109.471
O2—C6—N3	120.34 (9)	C8—C4—H4A	108.149
O2—C6—C7	124.67 (10)	C8—C4—H4B	108.145
N3—C6—C7	114.99 (9)	C10—C4—H4A	108.149
C6—C7—C8	121.47 (9)	C10—C4—H4B	108.153
C6—C7—C9	117.89 (9)	H4A—C4—H4B	107.318
C8—C7—C9	120.63 (9)	C7—C9—H9	120.087
C4—C8—C7	121.03 (9)	C11—C9—H9	120.091
C4—C8—C12	120.06 (9)	C9—C11—H11	120.082
C7—C8—C12	118.92 (10)	C13—C11—H11	120.074
C7—C9—C11	119.82 (10)	C8—C12—H12	119.713
O1—C10—N3	119.63 (9)	C13—C12—H12	119.710
O1—C10—C4	122.85 (10)	C11—C13—H13	119.902
N3—C10—C4	117.52 (9)	C12—C13—H13	119.895
			
H5—O5—N3—C6	150.2	N3—C6—C7—C9	−176.51 (8)
H5—O5—N3—C10	−30.7	C6—C7—C8—C4	0.00 (14)
O5—N3—C6—O2	−5.29 (13)	C6—C7—C8—C12	−179.84 (8)
O5—N3—C6—C7	174.61 (7)	C6—C7—C9—C11	−179.76 (8)
O5—N3—C10—O1	3.59 (14)	C6—C7—C9—H9	0.2
O5—N3—C10—C4	−176.79 (8)	C8—C7—C9—C11	0.67 (14)
C6—N3—C10—O1	−177.53 (9)	C8—C7—C9—H9	−179.3
C6—N3—C10—C4	2.09 (15)	C9—C7—C8—C4	179.56 (8)
C10—N3—C6—O2	175.80 (9)	C9—C7—C8—C12	−0.28 (14)
C10—N3—C6—C7	−4.30 (15)	C4—C8—C12—C13	179.79 (9)
C8—C4—C10—O1	−179.05 (9)	C4—C8—C12—H12	−0.2
C8—C4—C10—N3	1.35 (13)	C7—C8—C12—C13	−0.36 (15)
C10—C4—C8—C7	−2.26 (14)	C7—C8—C12—H12	179.6
C10—C4—C8—C12	177.58 (8)	C7—C9—C11—C13	−0.41 (15)
H4A—C4—C8—C7	119.8	C7—C9—C11—H11	179.6
H4A—C4—C8—C12	−60.4	H9—C9—C11—C13	179.6
H4B—C4—C8—C7	−124.3	H9—C9—C11—H11	−0.4
H4B—C4—C8—C12	55.5	C9—C11—C13—C12	−0.23 (16)
H4A—C4—C10—O1	58.9	C9—C11—C13—H13	179.8
H4A—C4—C10—N3	−120.7	H11—C11—C13—C12	179.8
H4B—C4—C10—O1	−57.0	H11—C11—C13—H13	−0.2
H4B—C4—C10—N3	123.4	C8—C12—C13—C11	0.62 (16)
O2—C6—C7—C8	−177.04 (9)	C8—C12—C13—H13	−179.4
O2—C6—C7—C9	3.39 (15)	H12—C12—C13—C11	−179.4
N3—C6—C7—C8	3.06 (13)	H12—C12—C13—H13	0.6

### Hydrogen-bond geometry (Å, º)

**Table:**

*D*—H···*A*	*D*—H	H···*A*	*D*···*A*	*D*—H···*A*
O5—H5···O1^i^	0.84	1.91	2.7056 (17)	158

Symmetry code: (i) −*x*+1, −*y*+1, −*z*+2.
